# The Reproducibility of Cerebrovascular Reactivity Across MRI Scanners

**DOI:** 10.3389/fphys.2021.668662

**Published:** 2021-05-06

**Authors:** Olivia Sobczyk, Ece Su Sayin, Kevin Sam, Julien Poublanc, James Duffin, Joseph A. Fisher, David J. Mikulis

**Affiliations:** ^1^Joint Department of Medical Imaging and the Functional Neuroimaging Laboratory, University Health Network, Toronto, ON, Canada; ^2^Department of Anaesthesia and Pain Management, University Health Network, Toronto, ON, Canada; ^3^Department of Physiology, University of Toronto, Toronto, ON, Canada; ^4^Department of Radiology, Johns Hopkins University School of Medicine, Baltimore, MD, United States; ^5^Institute of Medical Sciences, University of Toronto, Toronto, ON, Canada

**Keywords:** cerebrovascular reactivity, carbon dioxide, cerebral blood flow, MRI, reproducibility

## Abstract

Cerebrovascular reactivity (CVR) is defined as the ratio of the cerebral blood flow (CBF) response to an increase in a vasoactive stimulus. We used changes in blood oxygenation level-dependent (BOLD) MRI as surrogates for changes of CBF, and standardized quantitative changes in arterial partial pressure of carbon dioxide as the stimulus. Despite uniform stimulus and test conditions, differences in voxel-wise BOLD changes between testing sites may remain, attributable to physiologic and machine variability. We generated a reference atlas of normal CVR metrics (voxel-wise mean and SD) for each of two sites. We hypothesized that there would be no significant differences in CVR between the two atlases enabling each atlas to be used at any site. A total of 69 healthy subjects were tested to create site-specific atlases, with 20 of those individuals tested at both sites. 38 subjects were scanned at Site 1 (17F, 37.5 ± 16.8 y) and 51 subjects were tested at Site 2 (22F, 40.9 ± 17.4 y). MRI platforms were: Site 1, 3T Magnetom Skyra Siemens scanner with 20-channel head and neck coil; and Site 2, 3T HDx Signa GE scanner with 8-channel head coil. To construct the atlases, test results of individual subjects were co-registered into a standard space and voxel-wise mean and SD CVR metrics were calculated. Map comparisons of z scores found no significant differences between white matter or gray matter in the 20 subjects scanned at both sites when analyzed with either atlas. We conclude that individual CVR testing, and atlas generation are compatible across sites provided that standardized respiratory stimuli and BOLD MRI scan parameters are used. This enables the use of a single atlas to score the normality of CVR metrics across multiple sites.

## Introduction

The magnitude of cerebral hemodynamic responses to alterations in arterial partial pressure of CO_2_ (PaCO_2_), referred to as cerebrovascular reactivity (CVR), has increased understanding of the determinants of cerebral perfusion in many neurovascular disorders. Presently, there are a number of techniques that have been developed for the assessment and mapping of CVR. Of these, blood oxygenation level-dependent (BOLD) magnetic resonance imaging (MRI) signal intensity as a surrogate measure of cerebral blood flow (CBF) is in common use. However, the vasodilatory stimulus in most instances is uncontrolled, impeding test standardization, which is required for research and clinical use. Stimulus standardization requires precise, repeatable changes in PaCO_2_ independent of subject, or patient, efforts. A standardized “brain stress test” would provide a uniform platform from which to assess and manage a wide variety of disorders affecting brain blood flow regulation, such as in steno-occlusive disease (Brawley et al., 1967; Brawley, 1968; Symon, 1969; Kleiser and Widder, 1992; Yonas et al., 1993; Webster et al., 1995; Molina et al., 1999; Markus and Cullinane, 2001; Ogasawara et al., 2002; Sasoh et al., 2003; Mandell et al., 2008, 2011; Fierstra et al., 2010; Silvestrini et al., 2011; Sobczyk et al., 2016), glioma (Fierstra et al., 2018a; Sebok et al., 2020), arteriovenous malformations (Fierstra et al., 2011), traumatic brain injury (Da Costa et al., 2016; Mutch et al., 2016) and remote stroke effects (Sebok et al., 2018; Hendrik Bas van Niftrik et al., 2020; Sebok et al., 2021). The advancement of the clinical and research applications of CVR has required, first, the standardization of the method (Sobczyk et al., 2014; Sobczyk et al., 2015), and second, the verification of the repeatability of the test (Sobczyk et al., 2015; Sobczyk et al., 2016). The third and final step, and the aim of this paper, is to validate the compatibility of data between sites and across MR platforms.

Test variability between sites may arise from disease activity, equipment variability, artifacts such as patient movement and from normal day-to-day physiological variability. Performing CVR studies using a standard stimulus and a single MR scanner with consistent sequence parameters optimizes stimulus and machine repeatability (Kassner et al., 2010; Sobczyk et al., 2016); therefore observed variation of CVR metrics outside of test variability is more attributable to changes in physiology and disease states. However, as much as the BOLD MR signal is dependent on the MR pulse parameters, there may also be hardware dependent variability and thus may differ between vendors and platforms despite the same scan parameters.

The first step for comparing data across scanners required the generation of an atlas consisting of merged CVR data from healthy subjects from which was calculated a voxel-wise mean and standard deviation of CVR. Single subject’s CVR metrics can be compared to those of the atlas, analogous to the way blood clotting tests are compared to the normal range for *a particular* laboratory. Each voxel in an individual CVR scan would then be expressed in terms of standard deviations of the atlas mean (z score). Whereas absolute values for CVR may vary from center to center with the stimulus protocol and scan parameters, as long as the protocol for the CVR test and the atlas are the same, the z scores should be comparable across platforms. However, it would be very burdensome, expensive, and inefficient if an atlas had to be generated for every scanner. We hypothesized that if a repeatable and quantitative ventilatory protocol could be applied in conjunction with matching of critical MRI scan parameters, the variability in time-point to time-point and scanner-to-scanner variances could be minimized. A single healthy control atlas derived from one MRI system could then be used to assess single subjects across platforms. It would also enable the comparison of individuals and cohorts across institutions for use in multi-center trials.

The aim of this study was to evaluate whether the CVR atlases generated on different MRI platforms with the same, reproducible stimulus and the same scan parameters would have sufficient uniformity to be interchangeable across platforms for normalizing individual CVR data.

## Methods

### Subjects and Ethical Approval

This study conformed to the standards set by the latest revision of the Declaration of Helsinki and were approved by the Research Ethics Board of the University Health Network and Health Canada. All subjects provided written and informed consent to participate in this study. We recruited 69 voluntary, healthy participants (31 females) between the ages of 18–80 years old by advertisement and word of mouth. Each subject was in good health with no history of neurological or cardiovascular disease, non-smokers taking no medication. The demographics for the cohort are represented in Table 1.

**TABLE 1:** Summary of healthy subject demographics.

Age range	Scan at site 1 only	Scan at site 2 only	Scan at both sites
18–28	8	8	7
29–38	4	4	7
39–54	3	11	2
55–76	3	8	4
**Sex**			
F	9	14	8
M	9	17	12
Total	18	31	20

### Experimental Protocol

CVR studies were performed on two separate MRI platforms. At Site 1, a 3T Magnetom SKYRAfit Siemens scanner with a 20-channel head and neck coil (bore diameter of 70 cm, slew rate 200 T/m/s, gradient peak 45 mT/m, software version Syngo MR D13B) was used, while at Site 2, a 3T HDx Signa GE scanner with an 8-channel head coil (bore diameter of 60 cm, slew rate of 150 T/m/s, gradient peak of 50 mT/m, software version HD16.0_V02_1131.a) was used.

All CVR studies reported in this study at both Site 1 and Site 2 consisted of matching MRI parameters and identical experimental CO_2_ protocols. A total of 69 healthy subjects were scanned to create site-specific atlases (38 at Site 1 [17F, 37.5 ± 16.8 y] and 51 at Site 2 [22F, 40.9 ± 17.4 y]). From the 69 participants, 20 of those individuals completed CVRs at both sites within a 2-month time frame.

#### Hypercapnic Stimulus

**FIGURE 1:**
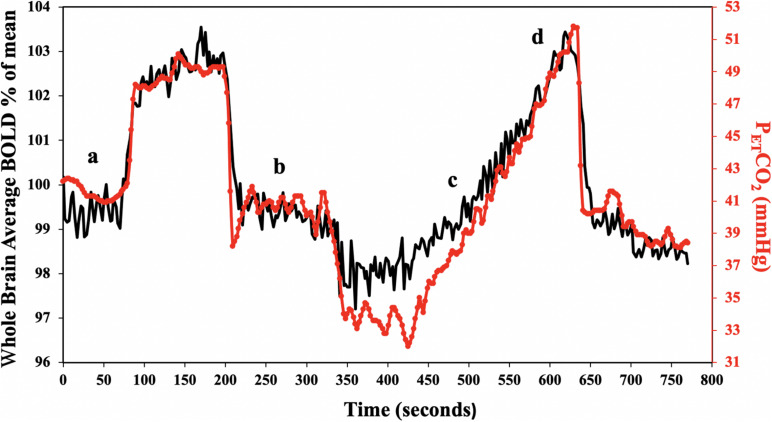
Standard P_ET_CO_2_ sequence in a representative subject. The CO_2_ stimulus (red line), and whole brain average BOLD response (black line) are shown. CVR was calculated for the step portion of the response **(a,b)**, and resting P_ET_CO_2_ + 10 mmHg ramp portion of the response **(c,d)**.

Subjects breathed through a fitted facemask connected to a sequential gas delivery breathing circuit (Somogyi et al., 2005). The CO_2_ stimulus consisted of clamping end-tidal partial pressure of CO_2_ (P_ET_CO_2_) at the subjects resting value for 2 min, followed by a step increase by 10 mmHg for 2 min and returned to baseline for another 2 min. P_ET_CO_2_ was then reduced by 10 mmHg for 1 minute, followed by a steady rise in P_ET_CO_2_ to 15 mmHg above baseline over ∼5 min, and returned to baseline for 2 min. The stimulus pattern shown in Figure 1 was programmed into a computer-controlled gas blender (RespirAct^TM^, Thornhill Research, Toronto, Canada) that runs a prospective gas targeting algorithm (Slessarev et al., 2007), which controls the CO_2_ stimulus independently of tidal volume and breathing frequency such that P_ET_CO_2_ is equivalent to PaCO_2_ (Ito et al., 2008; Willie et al., 2012). The entire CO_2_ stimulus was performed at normoxia (P_E__T_O2 ∼110 mmHg) that is also precisely controlled, independently of CO_2_, by the RespirAct^TM^ system.

#### Magnetic Resonance Imaging Protocol for Cerebrovascular Reactivity Map Generation

The protocol consisted of: **1.** 3D T1-weighted Inversion-Recovery Prepared Fast Spoiled Gradient Echo (IR-FSPGR) with TI = 450 ms, TR = 7.88 ms, TE = 3 ms, flip angle = 12°, voxel = 0.859 × 0.859 × 1 mm, matrix = 256 × 256, 146 slices, field of view = 24 × 24 cm, no interslice gap, acquisition time = 7.4 min. **2.** T2^∗^-weighted gradient recalled echo sequence with echo-planar readout (EPI-GRE) with TR = 2400 ms, TE = 30 ms, flip angle = 70°, 41 slices, voxel = 3.5 mm^3^, matrix = 64 × 64, number of volumes = 335, field of view = 24 × 24 cm, acquisition time = 13.4 min.

**FIGURE 2:**
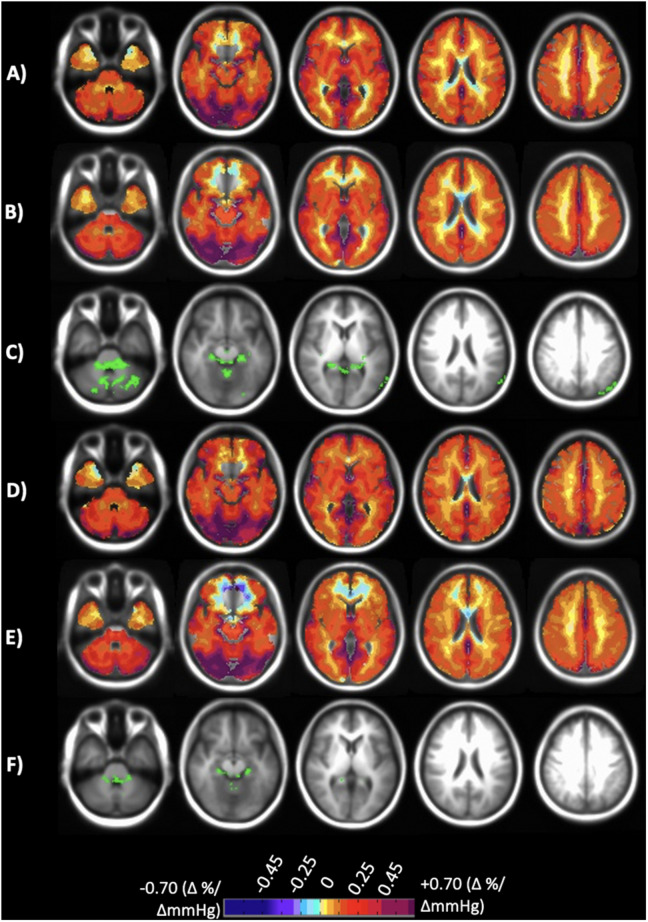
Axial slices of the normal cohort atlases calculated from only the individuals scanned at both sites. **(A)** Mean CVR values of step portion of the stimulus for the 20 individuals scanned at Site 1. **(B)** Mean CVR values of step portion of the stimulus for the 20 individuals scanned at Site 2. **(C)** Significant differences shown in green comparing A and B after multiple comparison correction *p* < 0.05. **(D)** Mean CVR values of the ramp (resting + 10 mmHg) portion of the stimulus for the 20 individuals scanned at Site 1. **(E)** Mean CVR values of ramp (resting + 10 mmHg) portion of the stimulus for the 20 individuals scanned at Site 2. **(F)** Significant differences shown in green comparing **(D,E)** after multiple comparison correction *p* < 0.05. CVR maps color-coded according to the scale shown on the bottom in percentage of BOLD change per mmHg P_ET_CO_2_ change.

The acquired BOLD MRI scan and P_ET_CO_2_ data were analyzed using AFNI software (National Institutes of Health, Bethesda, Maryland)^1^ (Cox, 1996). BOLD images were first volume registered and slice-time corrected and coregistered to the IR-FSPGR. P_ET_CO_2_ data was then time-shifted to the point of maximum correlation with the whole brain average BOLD signal. A linear, least-squares fit of the BOLD signal data series to the P_ET_CO_2_ data series was then performed on a voxel-by-voxel basis, together with a linear trend regressor to calculate CVR values. This method has been described in greater detail elsewhere (Fierstra et al., 2010; McKetton et al., 2018a). CVR for each dataset was calculated for different portions of the stimulus including the step portion of the stimulus (which includes dynamic changes in the CVR values (Poublanc et al., 2015), and the linear portion of the ramp stimulus starting from the individuals resting CO_2_ up to + 10 mmHg from resting (representing a steady state CVR). CVR was expressed as the percent change in BOLD signal per change in P_ET_CO_2_ (%/mmHg). Step and ramp CVR maps were color-coded based on a spectrum of colors denoting the magnitude of response in either the positive or negative direction (Figure 2).

### Analysis of Data

#### Construction of Cerebrovascular Reactivity Atlas

The normative data from all subjects across the two scanning sites were co-registered into Montreal Neurologic Institute (MNI) standard space using an analytical processing software (SPM8: Wellcome Department of Imaging Neuroscience, University of London, United Kingdom)^2^. A voxel-by-voxel mean CVR, and associated standard deviation (SD), was calculated for the step and ramp potions of the stimulus, to form site-specific atlases (AFNI software (Cox, 1996). Four sets of atlases were created for each of CVR parameters: atlases were created for subjects scanned at each site (38-person atlas at Site 1, 51-person atlas at Site 2), and atlases that included only those individuals that were scanned at both sites (20 person atlases for both Site 1 and Site 2). Atlas generation methodology has been described in greater detail elsewhere (Poublanc et al., 2015; Sobczyk et al., 2015; McKetton et al., 2018b). The co-registered T1-weighted anatomical images for all subjects were segmented into gray and white matter (GM and WM) using the aforementioned SMP8 software. These masks were used to calculated mean GM and WM values for each vascular territory by hemisphere [left (L) and right (R) MCA (middle cerebral artery), ACA (anterior cerebral artery) and PCA (posterior cerebral artery)] for the CVR step and ramp portion of the stimulus in each subject.

#### Statistical Analysis

Voxel-wise paired *t*-tests (α = 0.05) were used to determine if significant differences existed between the 20 person atlases of each CVR parameter (step and ramp), which contained only subjects that were scanned at both sites. Permutation testing was then applied to the height of the maxima of the resulting statistic image, using the “randomize” permutation-based inference tool (Winkler et al., 2014) in FSL v.5.0.9 (FMRIB Library^3^ that allowed for the maintenance of strong control over family-wise error. Additional statistical analyses were conducted on the mean GM, WM and hemispheric vascular territories for each sets of atlases using SigmaPlot 12.5 (Systat Software, Inc., San Jose California). To evaluate measurement reproducibility and investigate possible bias in the CVR data, Bland-Altman analysis was used to calculate the two sites limits of agreement between the 20 person atlases for GM and WM of each CVR parameter. A Two-Way repeated measures analysis of variance (rmANOVA) with the Holm-Sidak method of multiple comparison correction was performed on the 20 person atlases to determine if any significant differences existed between the groups (α = 0.05). For the full site atlases, a Two-Way ANOVA was performed with the Holm-Sidak method of multiple comparison correction (α = 0.05).

#### Construction of Z-Maps

Individualized z scores were calculated on a voxel-wise basis by co-registering the subject’s CVR map to the same space as the reference atlases followed by scoring the subject’s CVR values by assigning a z-score according to the mean and SD of the corresponding voxel of the atlas (Sobczyk et al., 2015). A healthy subject individual z-scores were calculated by a “leave one out”/“jackknife” procedure, which requires taking the healthy subject out from the atlases and z scoring that subject to atlases made up of the remaining subjects (Minoshima et al., 1995). The z values were then color coded and superimposed on the anatomical scans to form site-specific z-maps. Frequency distribution histograms (FDH) were created of the z-maps for each vascular territory. Absolute difference maps were calculated voxel-wise from the z-maps created from Site 1 and Site 2.

## Results

### Atlas Results for Only Subjects Scanned at Both Sites

CVR step and ramp atlas maps were created for the 20 individuals that were scanned at both sites and compared to one another to discern site-related differences. Figure 2 displays the atlas maps for CVR step and ramp at each site. A voxel-wise paired *t*-test for each of the CVR parameter maps between Site 1 and Site 2 was preformed, and significant differences (*p* < 0.05) are displayed in Figure 2. Minimal significant differences were found between Site 1 and Site 2 for both CVR parameters.

**FIGURE 3:**
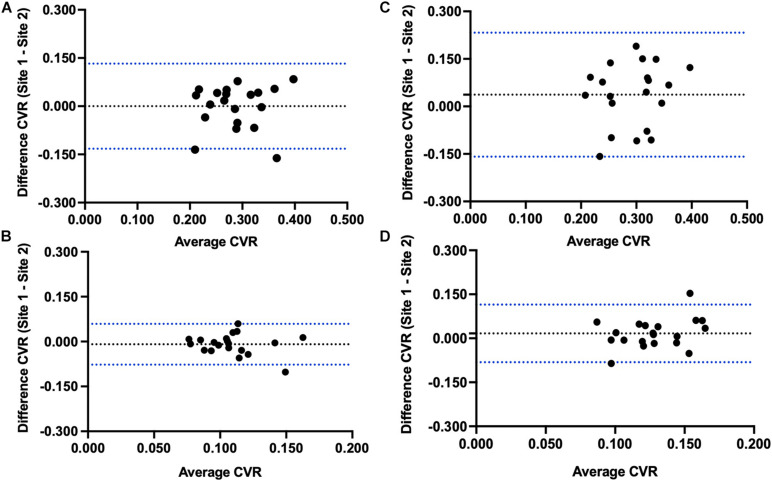
Bland-Altman plots comparing Site 1 and Site 2 differences in **(A)** step CVR gray matter, **(B)** step CVR white matter, **(C)** ramp CVR gray matter, and **(D)** ramp CVR white matter.

The results of the Bland-Altman analysis for between site reproducibility for the 20 individuals that were scanned at both sites are illustrated in Figure 3. Mean site difference for CVR step (mean ± 1.95 SD) was –1.15 × 10^–5^ ± 0.068%/mmHg in GM and –0.009 ± 0.035%/mmHg in WM. Mean site difference for CVR ramp was 0.037 ± 0.099%/mmHg in GM and 0.017 ± 0.050%/mmHg in WM.

Mean step and ramp CVR values of the 20 subjects scanned at Site 1 and Site 2 for GM, WM and each hemispheric vascular territory, were calculated. GM and WM values are presented in Table 2 along with the associated SD. Mean CVR values for each hemispheric vascular territory are presented in Supplementary Table 1. On average, CVR measures were similar in both GM and WM between sites (*P*-values presented in Table 2, α = 0.05). The Two-Way rmANOVA found a statistically significant interaction between site and region of interest (ROI) and found significant differences between sites for step CVR in the RACA GM and WM, and LPCA GM. Significant differences between sites for ramp CVR were found in the L and R MCA GM, and the LACA GM and WM (*P*-values presented in Supplementary Table 1, α = 0.05). An interpretation of these results with respect to the implications of the interaction between site and ROI factors is found in the discussion section.

**TABLE 2:** Mean, standard deviation (SD) and *p*-values for gray (GM) and white matter (WM) regions of each CVR parameter for the 20 healthy subjects that were scanned at both sites.

		Site 1		Site 2		
		Mean (%/mmHg)	SD	Mean (%/mmHg)	SD	*P*-value
CVR GM	Step	0.287	0.0649	0.287	0.0620	0.999
	Ramp	0.312	0.0781	0.275	0.0641	0.114
CVR WM	Step	0.104	0.0253	0.113	0.0310	0.255
	Ramp	0.136	0.0396	0.120	0.0280	0.154

### Atlas Results for All Subjects Scanned at Either Site

**FIGURE 4:**
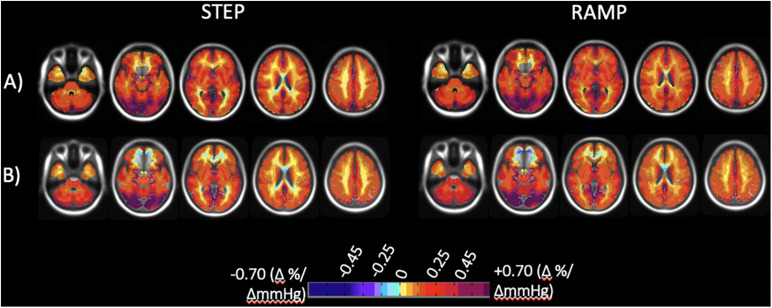
Axial slices of the normal cohort atlases calculated from all individuals for both the step and ramp (resting + 10 mmHg) portions of the stimulus. **(A)** Mean CVR values for all individuals scanned at Site 1 (38 individuals total). **(B)** Mean CVR values for all individuals scanned at Site 2 (51 individuals total). CVR maps color-coded according to the scale shown on the bottom in percentage of BOLD change per mmHg P_ET_CO_2_ change.

CVR step and ramp atlas maps were created for all subjects scanned at either site, with 38 subjects in total scanned at Site 1 and 51 subjects scanned at Site 2. Figure 4 displays the atlas maps for CVR step and ramp at Site 1 and Site 2.

A Two-Way ANOVA was performed for each of the CVR parameter maps comparing all subjects scanned at each site for GM, WM, and each hemispheric vascular territory. The overall means, SD, and associated *p*-values for GM and WM are presented in Table 3. Mean CVR values for each hemispheric vascular territory are presented in Supplementary Table 2. No significant difference between the CVR step and ramp for GM and WM were found between the two sites (*P*-values presented in Table 3, α = 0.05). For the hemispheric ROIs, a statistically significant interaction between site and region of interest (ROI) and significant differences between sites were found for step CVR in the L and R MCA GM, L and R ACA GM, LPCA GM, and L and R PCA WM. Significant differences between sites for ramp CVR were found in the LMCA GM, L and R ACA GM, L and R ACA WM, LPCA GM, and the L and R PCA WM (*P*-values presented in Supplementary Table 2, α = 0.05). An interpretation of these results with respect to the implications of the interaction between site and ROI factors is found in the discussion section.

**TABLE 3:** Mean, standard deviation (SD) and *p*-values for gray (GM) and white matter (WM) regions of each CVR parameter for all subjects that were scanned at each site.

		Site 1		Site 2		
		Mean (%/mmHg)	SD	Mean (%/mmHg)	SD	*P*-value
CVR GM	Step	0.300	0.0567	0.291	0.063	0.532
	Ramp	0.317	0.0669	0.303	0.071	0.337
CVR WM	Step	0.114	0.0278	0.116	0.032	0.901
	Ramp	0.143	0.0358	0.140	0.033	0.689

### Z-map Normalization Examples

**FIGURE 5:**
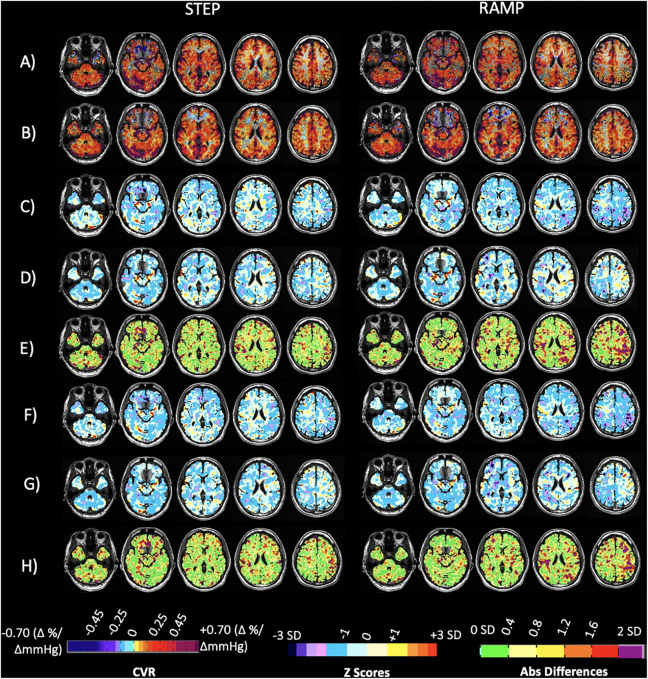
Case illustration of a normal subject. Displayed are axial slices for **(A)** the subject CVR maps for the step and ramp (resting P_ET_CO_2_ + 10 mmHg) portion of the stimulus obtained at Site 1. **(B)** The subject CVR maps for the step and ramp (resting P_ET_CO_2_ + 10 mmHg) portion of the stimulus obtained at Site 2. **(C)** Z-maps of the subject calculated by comparing their CVR parameter maps from Site 1 **(A)** to the corresponding 20 person CVR parameter atlases collected at Site 1. **(D)** Z-maps of the subject calculated by comparing their CVR parameter maps obtained at Site 2 **(B)** to the corresponding 20 person CVR parameter atlases collected at Site 2. **(E)** Voxel-wise absolute difference maps calculated from the z-maps in **(C,D)**. **(F)** Z-maps of the subject calculated by comparing their CVR parameter maps obtained from Site 1 **(A)** to the corresponding full 38 person CVR parameter atlases collected at Site 1. **(G)** Z-maps of the subject calculated by comparing their CVR parameter maps obtained from Site 2 **(B)** to the corresponding full 51 person CVR parameter atlases collected at Site 2. **(H)** Voxel-wise absolute difference maps calculated from the z-maps in F and G. Associated color scales found at bottom of figure. The CVR color scale denotes areas of positive and negative response in percentage of BOLD change per mmHg P_ET_CO_2_ change. The z scores and absolute difference scales provide a perspective of the statistically normal differences in CVR in standard deviation (SD).

Figure 5 presents the CVR and accompanying z-maps and absolute differences of the z scores from a healthy subject that was scanned at both sites and was removed from the atlases prior to calculating the z scores (known as a “jackknife” procedure what is used when taking a subject from a reference atlas and comparing them back to the atlas to perform z-scoring (Minoshima et al., 1995). The FDH of the step and ramp z scores for each vascular territory and an additional normal example is presented in Supplementary Material.

**FIGURE 6:**
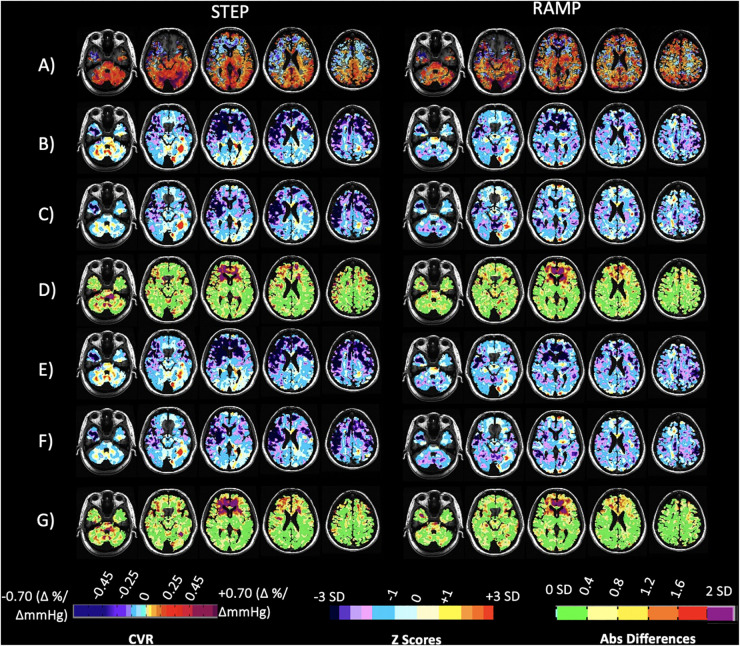
Case illustration of a 40-year-old patient with transient ischemic attacks and bilateral narrowing of the internal carotid arteries, scanned at Site 2 only. Displayed are axial slices for **(A)** the subject’s CVR maps for the step and ramp (resting P_ET_CO_2_ + 10 mmHg) portion of the stimulus. **(B)** Z-maps of the patient calculated by comparing their CVR parameter maps **(A)** to the corresponding 20 person CVR parameter atlases collected at Site 1. **(C)** Z-maps of the patient calculated by comparing their CVR parameter maps **(A)** to the corresponding 20 person CVR parameter atlases collected at Site 2. **(D)** Voxel-wise absolute difference maps calculated from the z-maps in **(B,C)**. **(E)** Z-maps of the patient calculated by comparing their CVR parameter maps in **(A)** to the corresponding full 38 person CVR parameter atlases collected at Site 1. **(F)** Z-maps of the patient calculated by comparing their CVR parameter maps in **(A)** to the corresponding full 51 person CVR parameter atlases collected at Site 2. **(G)** Voxel-wise absolute difference maps calculated from the z-maps in **(E,F)**. The CVR color scale denotes areas of positive and negative response in percentage of BOLD change per mmHg P_ET_CO_2_ change.

Figure 6 shows the application and comparison of the CVR atlases for each parameter to a patient with steno-occlusive disease whose CVR was collected at Site 2 only, 3 years after the atlas data was collected. Included are z scoring and absolute voxel-wise differences between the z-maps. The FDH of the step and ramp z scores for each vascular territory and an additional patient example with steno-occlusive disease is presented in Supplementary Material.

## Discussion

The main finding of this study is that voxel-wise CVR test results are not significantly different between two sites using different MR scanners when a precisely repeatable hypercapnic stimulus is applied and consistent MR scan parameters are specified. CVR test results of 20 subjects, scanned at both sites, were used to create atlases of CVR variability for the two locations. Voxel-wise paired *t*-tests applied to the atlases showed minimal differences (Figures 2C,F), and Bland-Altman analysis plots (Figure 3) displayed no proportional difference. Consequently, normalization of individual CVR values via z scores may be made using either atlas.

With respect to further atlas comparison using two-way rmANOVA, although such analysis of the step CVR atlases found significant differences for most of the GM and WM ACA territories (areas prone to susceptibility artifacts), these differences were not interpretable with respect to site difference since there was a statistically significant interaction between factors Site and ROI (*P* ≤ 0.001). The ramp CVR atlases which use only a few points of the full ramp stimulus (resting P_ET_CO_2_ + 10 mmHg) and are consequently more variable than the step CVR measurements, also showed several ROIs that were significantly different between sites, with the majority again in the ACA territories. However, interaction between site and ROI was also found and so these findings are not interpretable with respect to site difference.

Thus, an atlas of CVR in healthy people generated in one location can provide the same population normalization for CVR (Sobczyk et al., 2015) and interval differences in CVR (Sobczyk et al., 2016) regardless of where the individual measurements were made. Two patient examples (Figure 6 and Supplementary Figure 2) support this conclusion as well as the z score FDHs provided in Supplementary Material.

If the goal of using a single atlas to assess normality regardless of CVR testing site is to be achieved, every effort is required to eliminate non-physiologic variability. The two sources of CVR variability, stimulus and the measurement of its effect are the barriers to site-independent assessment of CVR. In studies where the vasoactive stimulus cannot be quantitated, much less standardized [e.g., acetazolamide injection, breath hold duration (Fierstra et al., 2013), and inhalation of a fixed CO_2_ concentration (Fisher, 2016)], it produces an unknowable variability in the CVR.

The CVR stimulus must be standardized, not only with respect to the sequence of CO_2_ changes used in terms of baseline, but also to the direction of change, amplitude, and duration of the true independent variable, the arterial PCO_2_. Giving a standard CVR stimulus that is comparable between patients, institutions, and over time, allows for the monitoring of a disease progress, and the results of therapeutic interventions to be assessed (Sobczyk et al., 2016) assuming inconsequential variability in the output of the MR scanner.

If the stimulus can be standardized the remaining source of technical variability is the variation in scanner settings. In the case of the atlases developed in this study, the main difference between the two platforms were the head coils. The Siemens scanner had a 20-channel coil whereas the GE scanner only had an 8-channel coil; however, the scan settings were the same. While this difference will affect the signal to noise ratio (SNR) between machines, the z-map images will be otherwise the same. The variability between subjects is expected to be greater than the experimental noise within a subject even with a lower SNR coil. The effect of using different scan parameters such as voxel size on the individual scans and comparing them to the atlases was not examined.

If these two technical issues are standardized, the remaining sources of variability between scans must arise from differences in physiological factors affecting the MR signals. These include the physiological expression of the variety of day-to-day experiences such as amount of sleep the previous night, foods eaten, tobacco use, emotional state, and ingested medications. However, the effects of these experiences may be dampened or cancel each other out. In any event, their effect size will be small compared to those of the technical conditions. Indeed test-retest differences were measured and quantified in a single scanner over weeks to months as mean ± SD for each voxel by Sobczyk et al. (2016). In the current study we show that CVR of normal individual voxels on 2 separate scanners fall within the mean and less than 2 SD of the CVR measured with either scanner.

### Clinical Implications

When z scoring of scans was first contemplated, it was considered that each institution would need to use their own standardized stimulus pattern for patients and repeat that pattern on a control group to generate an atlas for each scanner. Our findings suggest that for a standardized stimulus and MRI sequence, the variation in CVR is sufficiently small that a single atlas would be suitable across platforms. In addition, specialized atlases such as those controlling for sex, age, or medication, can be distributed across scanner locations. These considerations imply that all CVR data generated by a standard stimulus and scanner settings would be comparable, enabling a standard CVR test.

### Research Implications

For all intents and purposes, the CVR atlas functions as the “control” cohort for all CVR studies, thereby requiring smaller test cohorts. The reduced test variability would also enable the detection of smaller effect sizes with smaller cohorts. Data from various centers would be directly comparable, as is the case with other standardized tests such as the clotting test prothrombin time. The atlas can be made to have greater specificity for a given condition by narrowing the variability of the cohort to control for specific factors. For example, for examining CVR in patients with multiple sclerosis, the atlas can be restricted to subjects of younger age, and if examining females, then restricted by eliminating data from males.

### Limitations

Accurate measurements of CBF in advanced cerebrovascular disease is difficult to achieve. The limitations of BOLD as a surrogate measure of relative CBF changes are well known (Sobczyk et al., 2014; Duffin et al., 2015; Tancredi et al., 2015), nevertheless BOLD has been shown to correlate well with other modalities, including FAIR arterial spin-labeling (Hoge et al., 1999; Mandell et al., 2008; Chen and Pike, 2010) and more recently with (^15^O–)H_2_O positron emission tomography (PET) (Fierstra et al., 2018b; Hauser et al., 2019). With respect to the CO_2_ stimulus, although a general linear model (GLM) is assumed for matching BOLD to P_ET_CO_2_, this relationship is very complex and not necessarily well approximated by assuming a GLM (Fisher et al., 2017; Duffin et al., 2018).

Atlases were also constructed and compared between sites using all subjects tested so that they included CVR data from the 20 subjects studied in both locations. While this comparison is like that of the matched 20 atlases, nevertheless, we feel that these atlases were worth examining because they contained many subjects unique to a particular test site, and even when used to examine a cohort, the data from the overlapped subjects were gathered at the alternate location.

Small differences were apparent in both the atlas maps and the z score maps. The two scanner specific atlases derived from the same group of 20 individuals tested on both 3T scanners from different vendors were virtually identical but exhibited small differences (Figure 2, green in 2C and 2F). These were primarily within the cerebral spinal fluid of the ambient cisterns and cerebellum, which we attribute to susceptibility effects, and in venous structures. Similarly, when these atlases were derived from larger groups of different individuals, and included 20 of the same individuals, the atlases were virtually identical (Figure 4). However, there were differences in anterior inferior frontal lobe tissues. The BOLD signal is unreliable in areas where the applied static magnetic field is inhomogeneous. This typically occurs at the skull base and most often affects the anteromedial and inferior frontal lobes (gyrus rectus and orbito-frontal gyrus). CVR values in these regions are typically unreliable and can even be false positive for steal physiology.

Comparing CVR z score maps (Figures 6D,G) of a single patient calculated from control atlases generated from the same 20 healthy controls on each of the two scanners did find small differences after excluding those regions previously discussed where differences were presumably due to susceptibility effects. These differences were mostly within 0.4 SD, with a few regions showing 0.8 SD differences. Thus, a voxel-wise variance less than 1 SD is achievable when comparing a single individual against control atlases generated on different vendor scanners.

Variability within the atlases may be attributed to differences in age and sex, although we have not found differences in CVR across age ranges (McKetton et al., 2018a). Subjects for our reference CVR atlases were not selected for these or any other characteristics. As a result, the atlas contains a high variability in the normal cohort, which reduced the significance of the variability in output between platforms. However, the types of vascular disease typically studied including moyamoya, steno-occlusive disease, small vessel disease, and traumatic brain injury, result in large CVR changes that exceed those of machine variability, and thus a single atlas is suitable for these conditions. Should very subtle CVR changes be sought, the method may be made more sensitive by generating a local atlas with a cohort that contains the characteristics and medical conditions, or medications, for which one wishes to control; for example, assembling a cohort of women between the ages of 20 and 30 in an atlas for investigation for subtle CVR signs in multiple sclerosis.

## Conclusion

This study has demonstrated the feasibility of using a single atlas across platforms. A single atlas would be useful for normalizing clinical studies of individual patients as well as pooling compatible data in multicenter trials.

## Data Availability Statement

Anonymized data will be shared by request from any qualified investigator for purposes such as replicating procedures and results presented in the article provided that data transfer is in agreement with the research institution on the general data protection regulation. Requests to access the datasets should be directed to JAF, joe.fisher@utoronto.ca

## Ethics Statement

The studies involving human participants were reviewed and approved by the University Health Network. The patients/participants provided their written informed consent to participate in this study. Written informed consent was obtained from the individual(s) for the publication of any potentially identifiable images or data included in this article.

## Author Contributions

OS and KS were involved with data collection. OS, ES, JD, and JAF were involved in the data analysis and drafted the manuscript. All authors participated in the feedback and writing process following the initial drafting of the manuscript, contributed to the design and conceptualization of the study.

## Conflict of Interest

JAF and DM contributed to the development of the automated end-tidal targeting device, RespirAct^TM^ (Thornhill Research Inc., TRI) used in this study and have equity in the company. OS and JD received salary support from TRI. TRI provided no other support for the study. The remaining authors declare that the research was conducted in the absence of any commercial or financial relationships that could be construed as a potential conflict of interest.

## Footnotes

1 http://afni.nimh.nih.gov/afni

2 http://www.fil.ion.ucl.ac.uk/spm/software/spm8

3 https://fsl.fmrib.ox.ac.uk/fsl/fslwiki/
