# Supplementary material for: The Reproducibility of Cerebrovascular Reactivity Across MRI Scanners
Source: Front Physiol. 2021 May 6;12:668662. doi: 10.3389/fphys.2021.668662 (PMC8134667; doi:10.3389/fphys.2021.668662)
Supplement: Supplementary file 1 [file Data_Sheet_1.docx]

**Supplemental Material**


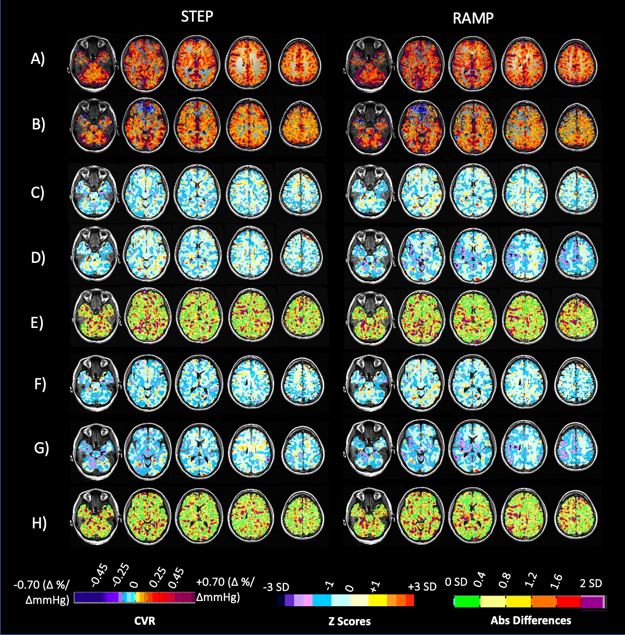


**Supplemental Figure 1.** Additional case illustration of a normal subject. Displayed are axial slices for A) the subject CVR maps for the step and ramp (resting P_ET_CO_2_+10mmHg) portion of the stimulus obtained at Site 1. B) The subject CVR maps for the step and ramp (resting P_ET_CO_2_+10mmHg) portion of the stimulus obtained at Site 2. C) Z-maps of the subject calculated by comparing their CVR parameter maps from Site 1 (A) to the corresponding 20 person CVR parameter atlases collected at Site 1. D) Z-maps of the subject calculated by comparing their CVR parameter maps obtained at Site 2 (B) to the corresponding 20 person CVR parameter atlases collected at Site 2. E) Voxel-wise absolute difference maps calculated from the z-maps in C and D. F) Z-maps of the subject calculated by comparing their CVR parameter maps obtained from Site 1 (A) to the corresponding full 38 person CVR parameter atlases collected at Site 1. G) Z-maps of the subject calculated by comparing their CVR parameter maps obtained from Site 2 (B) to the corresponding full 51 person CVR parameter atlases collected at Site 2. H) Voxel-wise absolute difference maps calculated from the z-maps in F and G. Associated color scales found at bottom of figure. The CVR color scale denotes areas of positive and negative response in percentage of BOLD change per mmHg P_ET_CO_2_ change. The z scores and absolute difference scales provide a perspective of the statistically normal differences in CVR in standard deviation (SD).

**
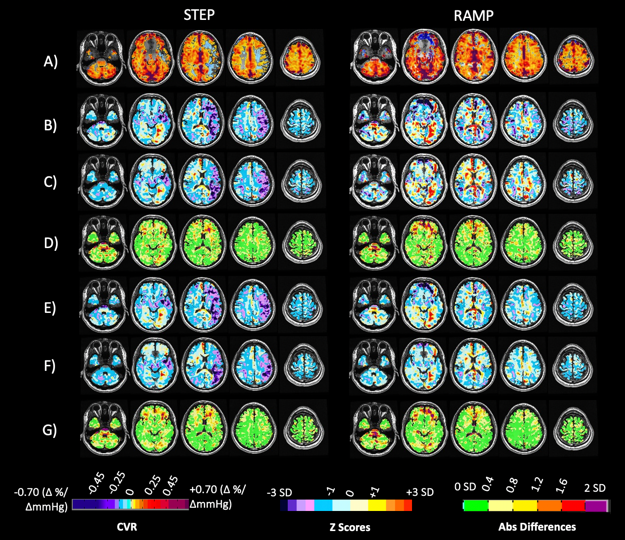
**

**Supplemental Figure 2.**  Additional case illustration of a 32-year-old patient with a left middle cerebral artery occlusion, scanned at Site 2 only. Displayed are axial slices for A) the subject’s CVR maps for the step and ramp (resting P_ET_CO_2_+10mmHg) portion of the stimulus. B) Z-maps of the patient calculated by comparing their CVR parameter maps (A) to the corresponding 20 person CVR parameter atlases collected at Site 1. C) Z-maps of the patient calculated by comparing their CVR parameter maps (A) to the corresponding 20 person CVR parameter atlases collected at Site 2. D) Voxel-wise absolute difference maps calculated from the z-maps in B and C. E) Z-maps of the patient calculated by comparing their CVR parameter maps in (A) to the corresponding full 38 person CVR parameter atlases collected at Site 1. F) Z-maps of the patient calculated by comparing their CVR parameter maps in (A) to the corresponding full 51 person CVR parameter atlases collected at Site 2. G) Voxel-wise absolute difference maps calculated from the z-maps in E and F. The CVR color scale denotes areas of positive and negative response in percentage of BOLD change per mmHg P_ET_CO_2_ change.

**
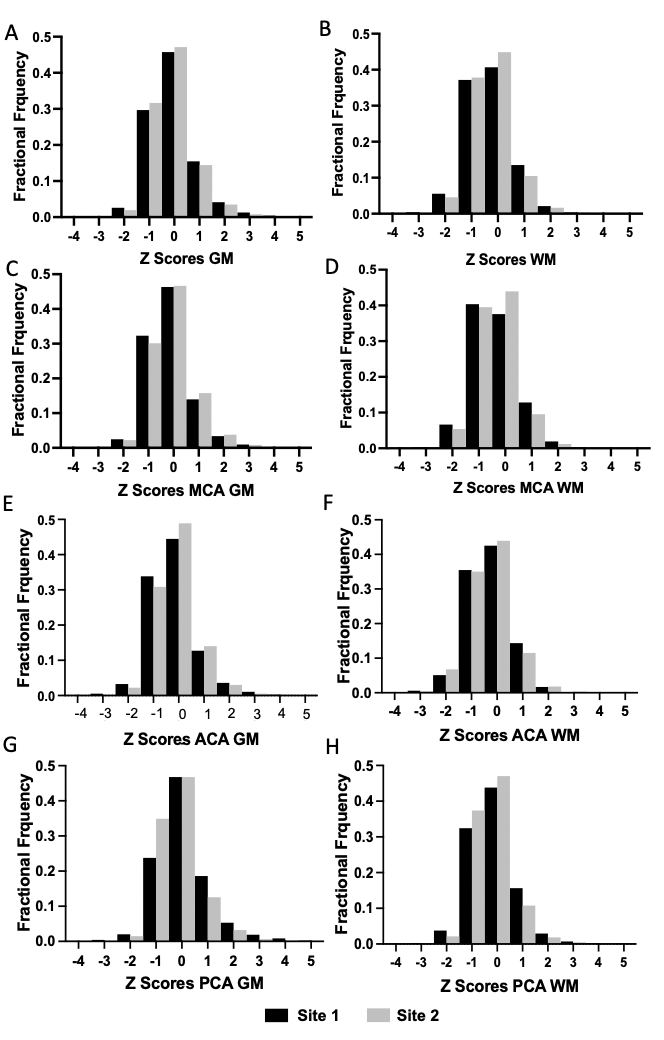
**

**Supplemental Figure 3.** CVR step z score FDH of GM, WM and vascular territories for Site 1 and Site 2 of the normal case illustration found in Figure 5 of the main paper, calculated by comparing their step CVR maps from each site to the corresponding 20 person step CVR site atlas (Figure 5C,D). ACA, anterior cerebral artery; CVR, cerebrovascular reactivity; FDH, frequency distribution histogram; GM, gray matter; MCA, middle cerebral artery; PCA, posterior cerebral artery; WM, white matter.

**
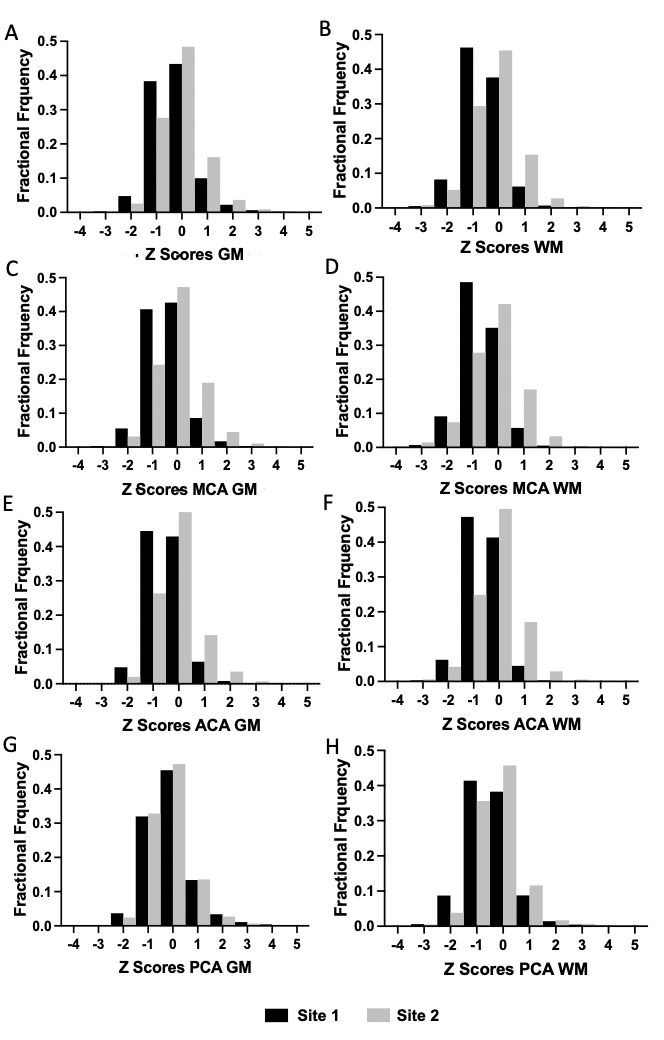
**

**Supplemental Figure 4.** CVR ramp z score FDH of GM, WM and vascular territories for Site 1 and Site 2 of the normal case illustration found in Figure 5 of the main paper, calculated by comparing their ramp CVR maps from each site to the corresponding 20 person ramp CVR site atlas (Figure 5C,D). ACA, anterior cerebral artery; CVR, cerebrovascular reactivity; FDH, frequency distribution histogram; GM, gray matter; MCA, middle cerebral artery; PCA, posterior cerebral artery; WM, white matter.

**
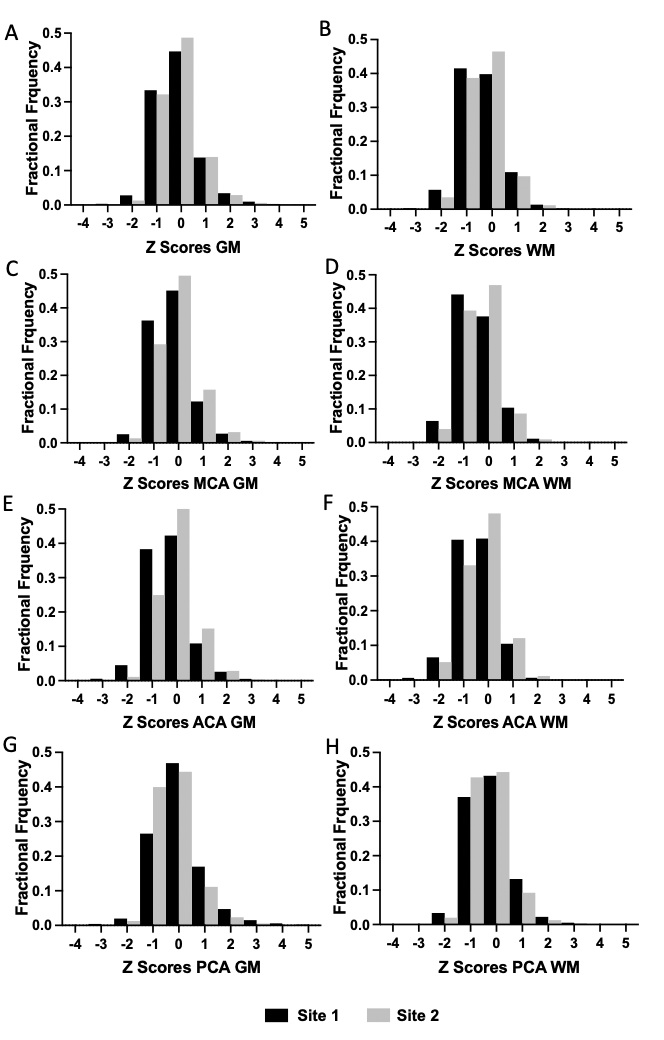
**

**Supplemental Figure 5.** CVR step z score FDH of GM, WM and vascular territories for Site 1 and Site 2 of the normal case illustration found in Figure 5 of the main paper, calculated by comparing their step CVR maps from each site to the corresponding full step CVR site atlas (Figure 5F,G). ACA, anterior cerebral artery; CVR, cerebrovascular reactivity; FDH, frequency distribution histogram; GM, gray matter; MCA, middle cerebral artery; PCA, posterior cerebral artery; WM, white matter.

**
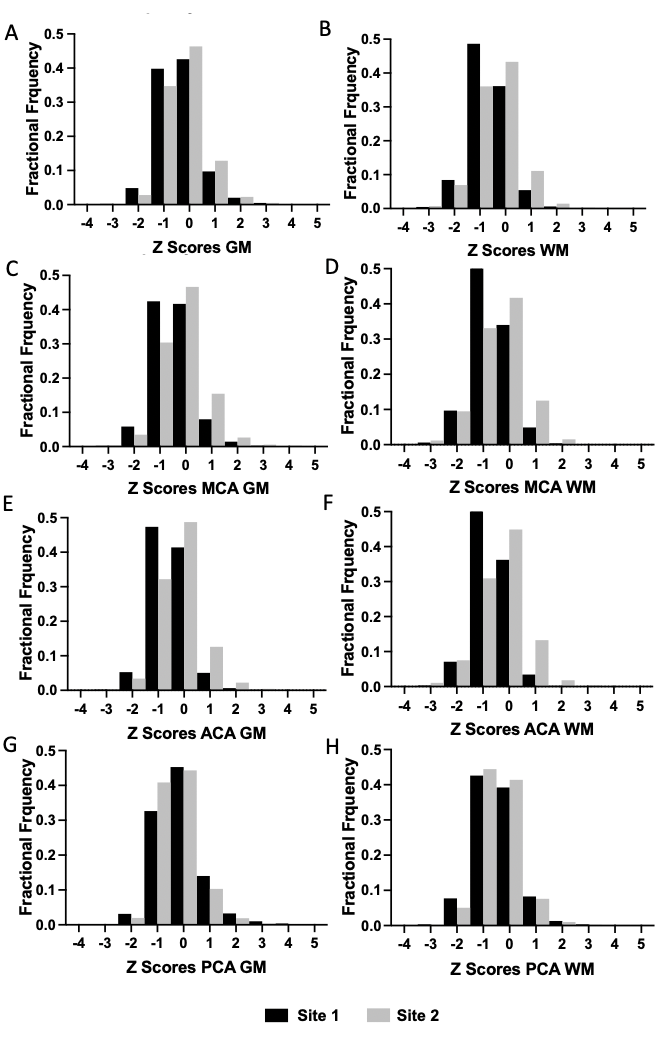
**

**Supplemental Figure 6.** CVR ramp z score FDH of GM, WM and vascular territories for Site 1 and Site 2 of the normal case illustration found in Figure 5 of the main paper, calculated by comparing their ramp CVR maps from each site to the corresponding full ramp CVR site atlas (Figure 5F,G). ACA, anterior cerebral artery; CVR, cerebrovascular reactivity; FDH, frequency distribution histogram; GM, gray matter; MCA, middle cerebral artery; PCA, posterior cerebral artery; WM, white matter.

**
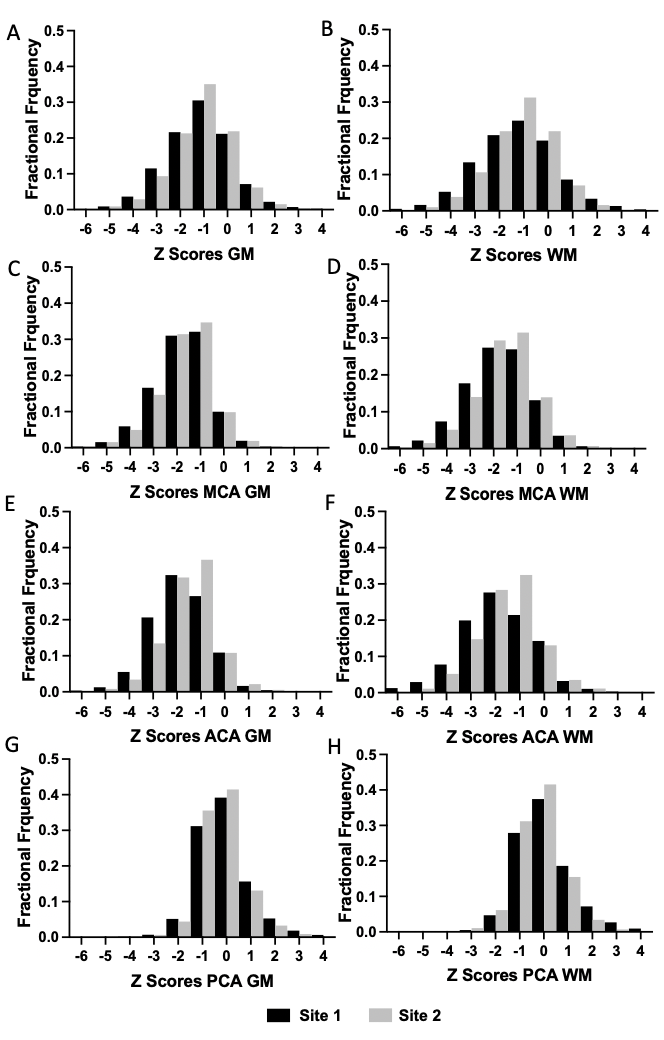
**

**Supplemental Figure 7.** CVR step z score FDH of GM, WM and vascular territories for Site 1 and Site 2 of the patient case illustration in Figure 6 of the main paper, calculated by comparing their step CVR map from Site 2 to the 20 person step CVR site atlases (Figure 6B,C). ACA, anterior cerebral artery; CVR, cerebrovascular reactivity; FDH, frequency distribution histogram; GM, gray matter; MCA, middle cerebral artery; PCA, posterior cerebral artery; WM, white matter.

**
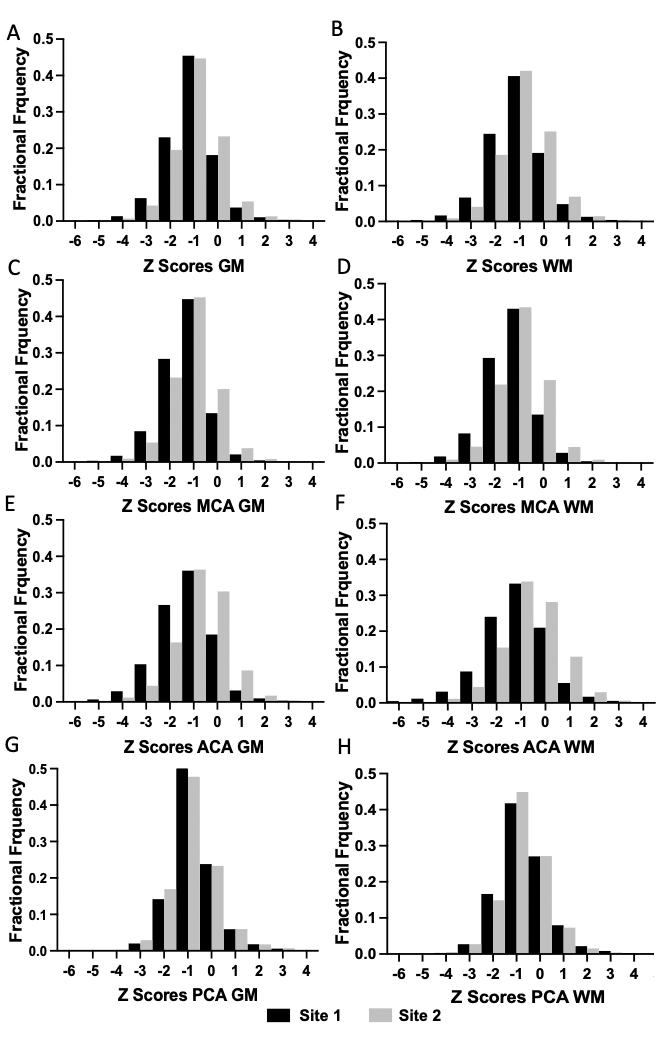
**

**Supplemental Figure 8.** CVR ramp z score FDH of GM, WM and vascular territories for Site 1 and Site 2 of the patient case illustration in Figure 6 of the main paper, calculated by comparing their ramp CVR map from Site 2 to the 20 person ramp CVR site atlases (Figure 6B,C). ACA, anterior cerebral artery; CVR, cerebrovascular reactivity; FDH, frequency distribution histogram; GM, gray matter; MCA, middle cerebral artery; PCA, posterior cerebral artery; WM, white matter.

**
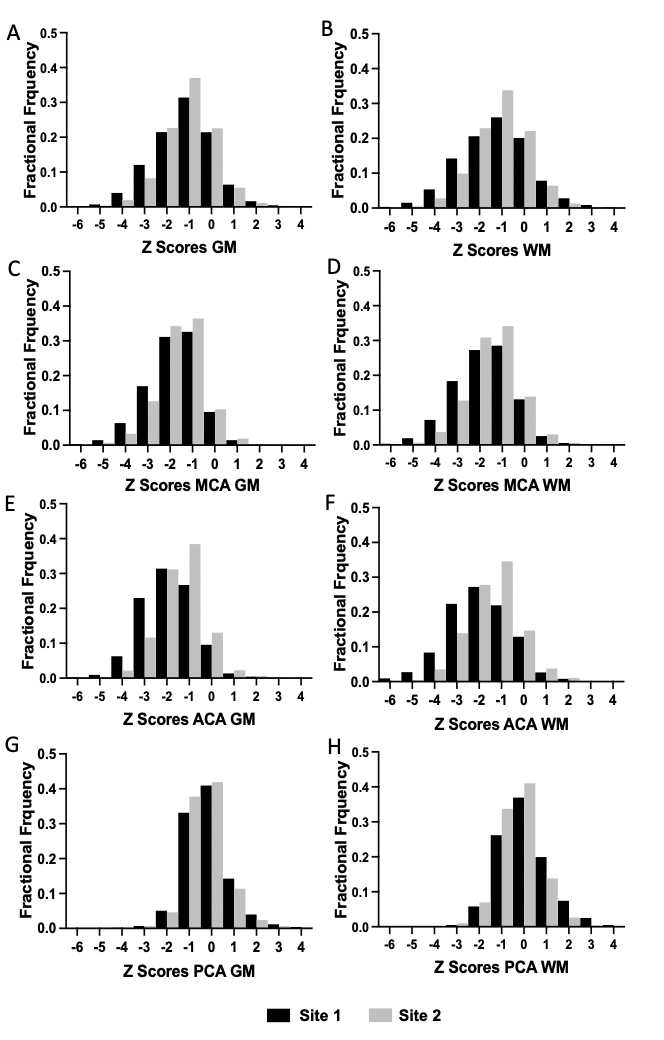
**

**Supplemental Figure 9.** CVR step z score FDH of GM, WM and vascular territories for Site 1 and Site 2 of the patient case illustration in Figure 6 of the main paper, calculated by comparing their step CVR map from Site 2 to the full step CVR site atlases (Figure 6E,F). ACA, anterior cerebral artery; CVR, cerebrovascular reactivity; FDH, frequency distribution histogram; GM, gray matter; MCA, middle cerebral artery; PCA, posterior cerebral artery; WM, white matter.

**
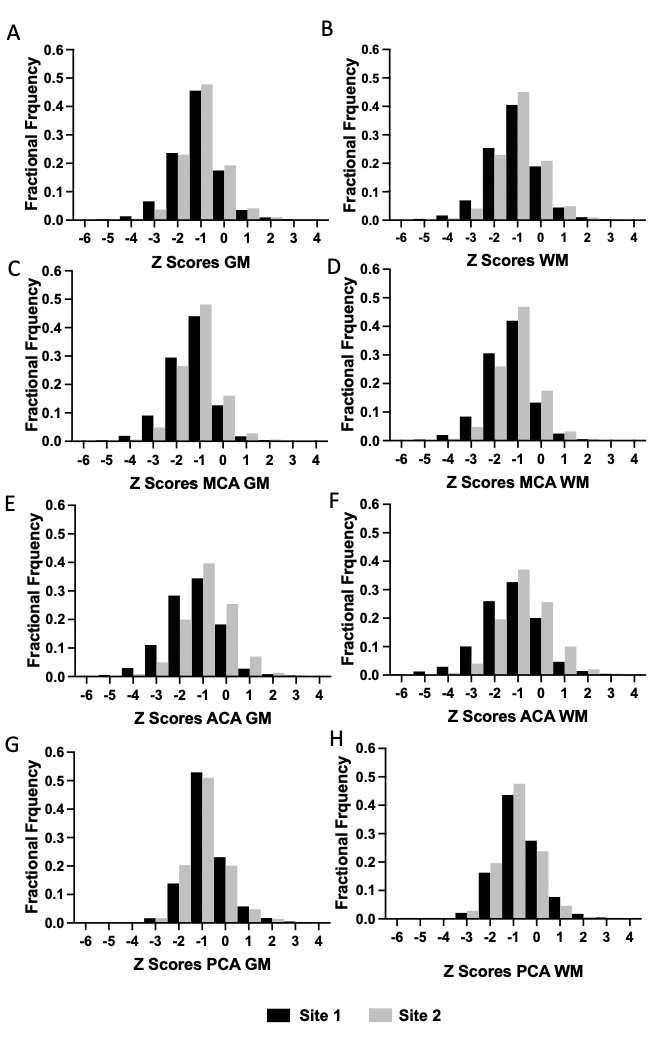
**

**Supplemental Figure 10.** CVR ramp z score FDH of GM, WM and vascular territories for Site 1 and Site 2 of the patient case illustration in Figure 6 of the main paper, calculated by comparing their ramp CVR map from Site 2 to the full ramp CVR site atlases (Figure 6E,F). ACA, anterior cerebral artery; CVR, cerebrovascular reactivity; FDH, frequency distribution histogram; GM, gray matter; MCA, middle cerebral artery; PCA, posterior cerebral artery; WM, white matter.

**Supplemental Table 1.**  Mean, standard deviation (SD) and p-values of each CVR parameter per hemispheric vascular territory for the 20 healthy subjects that were scanned at both sites. ACA, anterior cerebral artery; CVR, cerebrovascular reactivity; GM, gray matter; L, Left; MCA, middle cerebral artery; PCA, posterior cerebral artery; R, Right; WM, white matter. * indicates significant differences (*α* = 0.05).

|  |  | **Site 1** | | **Site 2** | |  |
| --- | --- | --- | --- | --- | --- | --- |
|  |  | **mean (%/mmHg)** | **SD** | **mean (%/mmHg)** | **SD** | **P-value** |
| **CVR LMCA GM** | **Step** | 0.256 | 0.0595 | 0.246 | 0.0545 | 0.508 |
|  | **Ramp** | 0.273 | 0.0780 | 0.223 | 0.0615 | 0.022* |
| **CVR LMCA WM** | **Step** | 0.099 | 0.0242 | 0.101 | 0.0274 | 0.877 |
|  | **Ramp** | 0.273 | 0.0780 | 0.109 | 0.0296 | 0.223 |
| **CVR RMCA GM** | **Step** | 0.265 | 0.0587 | 0.243 | 0.0504 | 0.141 |
|  | **Ramp** | 0.266 | 0.0707 | 0.218 | 0.0594 | 0.028* |
| **CVR RMCA WM** | **Step** | 0.100 | 0.0264 | 0.100 | 0.0293 | 0.987 |
|  | **Ramp** | 0.128 | 0.0413 | 0.108 | 0.0248 | 0.353 |
| **CVR LACA GM** | **Step** | 0.229 | 0.0759 | 0.225 | 0.0803 | 0.791 |
|  | **Ramp** | 0.252 | 0.0884 | 0.173 | 0.0923 | <0.001* |
| **CVR LACA WM** | **Step** | 0.064 | 0.0319 | 0.061 | 0.0395 | 0.834 |
|  | **Ramp** | 0.099 | 0.0548 | 0.047 | 0.0482 | 0.017* |
| **CVR RACA GM** | **Step** | 0.241 | 0.0908 | 0.237 | 0.0881 | <0.001* |
|  | **Ramp** | 0.264 | 0.0943 | 0.187 | 0.1031 | 0.051 |
| **CVR RACA WM** | **Step** | 0.064 | 0.0333 | 0.060 | 0.0383 | <0.001* |
|  | **Ramp** | 0.982 | 0.0420 | 0.051 | 0.0494 | 0.031* |
| **CVR LPCA GM** | **Step** | 0.335 | 0.0673 | 0.370 | 0.0807 | 0.023* |
|  | **Ramp** | 0.381 | 0.0891 | 0.390 | 0.0816 | 0.681 |
| **CVR LPCA WM** | **Step** | 0.131 | 0.0255 | 0.160 | 0.0419 | 0.054 |
|  | **Ramp** | 0.167 | 0.0378 | 0.181 | 0.0435 | 0.513 |
| **CVR RPCA GM** | **Step** | 0.379 | 0.0950 | 0.384 | 0.0818 | 0.717 |
|  | **Ramp** | 0.424 | 0.1049 | 0.408 | 0.0747 | 0.469 |
| **CVR RPCA WM** | **Step** | 0.141 | 0.0288 | 0.169 | 0.0450 | 0.062 |
|  | **Ramp** | 0.172 | 0.0339 | 0.191 | 0.0435 | 0.385 |

**Supplemental Table 2.** Mean, standard deviation (SD) and p-values of each CVR parameter per hemispheric vascular territory for all subjects that were scanned at each site. ACA, anterior cerebral artery; CVR, cerebrovascular reactivity; GM, gray matter; L, Left; MCA, middle cerebral artery; PCA, posterior cerebral artery; R, Right; WM, white matter. * indicates significant differences (*α* = 0.05).

|  |  | **Site 1** | | **Site 2** | |  |
| --- | --- | --- | --- | --- | --- | --- |
|  |  | **mean (%/mmHg)** | **SD** | **mean (%/mmHg)** | **SD** | **P-value** |
| **CVR LMCA GM** | **Step** | 0.270 | 0.0551 | 0.242 | 0.060 | 0.019* |
|  | **Ramp** | 0.282 | 0.0710 | 0.242 | 0.069 | 0.004* |
| **CVR LMCA WM** | **Step** | 0.109 | 0.0332 | 0.100 | 0.029 | 0.443 |
|  | **Ramp** | 0.144 | 0.0466 | 0.125 | 0.035 | 0.162 |
| **CVR RMCA GM** | **Step** | 0.273 | 0.0500 | 0.246 | 0.055 | 0.029* |
|  | **Ramp** | 0.270 | 0.0624 | 0.245 | 0.066 | 0.071 |
| **CVR RMCA WM** | **Step** | 0.106 | 0.0262 | 0.102 | 0.030 | 0.704 |
|  | **Ramp** | 0.132 | 0.0366 | 0.127 | 0.032 | 0.725 |
| **CVR LACA GM** | **Step** | 0.247 | 0.0670 | 0.215 | 0.077 | 0.008* |
|  | **Ramp** | 0.263 | 0.0809 | 0.202 | 0.088 | <0.001* |
| **CVR LACA WM** | **Step** | 0.076 | 0.0360 | 0.058 | 0.041 | 0.133 |
|  | **Ramp** | 0.114 | 0.0509 | 0.069 | 0.047 | 0.002* |
| **CVR RACA GM** | **Step** | 0.257 | 0.0758 | 0.230 | 0.089 | 0.027* |
|  | **Ramp** | 0.273 | 0.0821 | 0.219 | 0.098 | <0.001* |
| **CVR RACA WM** | **Step** | 0.076 | 0.0316 | 0.059 | 0.044 | 0.164 |
|  | **Ramp** | 0.105 | 0.0388 | 0.073 | 0.046 | 0.021* |
| **CVR LPCA GM** | **Step** | 0.350 | 0.0636 | 0.380 | 0.080 | 0.014* |
|  | **Ramp** | 0.388 | 0.0783 | 0.418 | 0.094 | 0.034* |
| **CVR LPCA WM** | **Step** | 0.141 | 0.0318 | 0.167 | 0.038 | 0.030* |
|  | **Ramp** | 0.173 | 0.1742 | 0.203 | 0.043 | 0.034* |
| **CVR RPCA GM** | **Step** | 0.385 | 0.0823 | 0.404 | 0.088 | 0.126 |
|  | **Ramp** | 0.419 | 0.0881 | 0.448 | 0.095 | 0.070 |
| **CVR RPCA WM** | **Step** | 0.147 | 0.0305 | 0.182 | 0.041 | 0.005* |
|  | **Ramp** | 0.174 | 0.0331 | 0.218 | 0.048 | 0.002* |
